# Evaluating the Comprehensive Benefit of Urban Renewal Projects on the Area Scale: An Integrated Method

**DOI:** 10.3390/ijerph20010606

**Published:** 2022-12-29

**Authors:** Yizhong Chen, Guiwen Liu, Taozhi Zhuang

**Affiliations:** 1 Civil Engineering Department, Yancheng Institute of Technology, Yancheng 224051, China; 2 School of Management Science & Real Estate, Chongqing University, Chongqing 400045, China

**Keywords:** urban renewal, area scale, comprehensive benefit evaluation, entropy weight method, back propagation neural network

## Abstract

Globally, the challenges facing cities regarding urban decay, insufficient urban function, and fragmented urban development are enormous. Under this context, urban renewal provides opportunities to address these challenges and enhance urban sustainability. Thus, promoting urban renewal projects and improving their performance is a global topic. In many circumstances, urban renewal is planned and initiated on the project scale, but on the area scale, overall coordination of the projects can bring about comprehensive benefits to urban areas on a macro view. In practice, it still lacks a systematic evaluation approach to obtain a clear picture of such comprehensive benefits. In academia, the existing research studies are mainly focused on single-project evaluation. An integrated framework that provides a holistic assessment of area-scale project benefits is missing. Few fully consider the coupling coordination benefits between several urban renewal projects from an area-scale perspective. Thus, this paper aims to propose a framework for integrating an indicator evaluation system through a hybrid entropy weight method with Back Propagation (BP) neural network methods to evaluate the comprehensive benefit of urban renewal projects on the area scale, which is the level at which most development area-scale renewal projects take place in a city. The feasibility and effectiveness of this proposed framework are then verified in a case study of Chongqing, China. The results indicate that the proposed method that integrated multi-project characteristics can contribute to a bigger picture of benefit evaluation of urban renewal based on an area scale perspective. This therefore provides not only guidance for urban planners and policymakers to make better decisions, but also new insight for benefit evaluation in the field of urban development.

## 1. Introduction

Globally, urban development has posed tremendous pressure on the use pattern of existing urban land [1,2]. The existing land function cannot match the citizens’ increasing rigid demands. In contrast, extensive land expansion and new construction in the urban built-up areas have challenged the urban carrying capacity [3,4]. Within this context, urban renewal has become a primary driving force in solving multiple urban development problems worldwide [5,6], and it intends to improve the environment of urban built-up areas according to people’s changing social and economic requirements [7,8].

In many cases, there are still significant challenges in carrying out urban renewal projects. There are several forms of urban renewal, mainly including redevelopment and rehabilitation. Redevelopment can effectively attract market investment, but demolition and reconstruction have been criticized for causing severely unsustainable problems [9]. Rehabilitation does not demolish the existing buildings and can better meet the goal of sustainability, yet it provides significantly fewer market values for the investors to participate in [10]. Nonetheless, the rehabilitation-type project is one of the major types of urban renewal. For instance, in 2019, there were approximately 160,000 old and dilapidated neighborhoods with a building area of 800 million m^2^ needing to be rehabilitated, influencing more than 42 million families in China [11,12]. Huge barriers exist for these large amounts of low or non-profitable projects to be successfully implemented due to insufficient public resources and their weak attraction to the private sector [13].

Under this context, the overall coordination of urban renewal projects in the area scale is proposed as the effective solution to solve this problem [14,15]. In a specific area, the highly profitable components (e.g., redevelopment or operational projects) can be bundled with low or unprofitable components (e.g., rehabilitation projects) to fulfill the requirement of financial balance. It improves sustainability and revitalizes the old city via integrally arranging and designating urban renewal projects on the area scale [15]. In this way, the profits of some components can be transferred to cover the expenses of others [16]. In addition, compared to the single project view, planning projects from the area-scale view can benefit cost saving, profit increase, and efficiency improvement [15]. Today, the idea of area-scale urban renewal has been proposed in many countries concerning both project feasibility and sustainable urban development [17]. For example, in the UK, Germany, and France, district-wide approaches have been advocated for that focus not only on individual historic buildings but also on the urban fabric as a whole [15]. The performance of urban renewal on the area scale is not only directly related to the sustainability of urban built-up areas but also refers to the planning of future multi-project development strategies [18].

With the growing concern of area-scale initiation and implementation, which urban renewal projects on the area scale are performing well remains to be a significant concern. In Chongqing, China, more than 60% of urban renewal projects have been conducted in the way of overall coordination on the area scale. It has been acknowledged that early, continuous, and cautious evaluation of project performance can provide a clear picture of current project implementation, which may facilitate the development of appropriate strategies [19,20]. The practical demands require a method for evaluating the comprehensive benefits of urban renewal projects on the area scale to provide a foundation and guidance for urban planners and policymakers.

Existing studies have investigated the benefits and impacts of urban renewal at the single-project or city level [5,14,21]. However, there is a lack of area-scale renewal project performance assessments from a macro perspective. In addition, most research regarding urban renewal performance evaluation methods involves many single-model evaluations, which strongly rely on the subjective opinions of specific experts or groups [14,22,23]. These methods can only be used in a specific context and cannot provide a holistic view of decision-making in this urban development era [24]. Furthermore, it lacks systematic guidance for the appropriate indicator list of urban renewal project evaluations on the area scale. Some relevant studies in the field of urban studies exist, but they all focus on newly built projects [25,26]. The indicators they adopt cannot match the salient features of urban renewal.

Therefore, to compensate for these shortcomings, this study aims to develop a method for evaluating the comprehensive benefits of urban renewal projects on the area scale. It proposes a framework that integrates a sustainability indicator and coupling coordination indicator evaluation system and innovatively designs a hybrid approach that combines the entropy weight method with BP neural network for comprehensive benefit evaluations to obtain more convincing quantitative results. Thus, this study enriches urban renewal performance assessment literature by making significant contributions. First, this research identified the unique criteria for area-scale renewal performance evaluation based on the extant literature review, especially considering the coupling coordination benefits between the multiple projects. Second, this study used the entropy weight method and BP neural network to determine the final weight value for each criterion. Compared to the traditional single evaluation method, the primary advantage of this integrated method is that it can make use of the advantages of each method and make up for each other’s deficiencies to reduce the deviation of evaluation results. The proposed conceptual framework in this study is also applicable to other types of urban development projects.

The remainder of this paper is organized as follows: it reviews the literature regarding the overall coordination of urban renewal on the area scale, the benefit evaluation of urban renewal projects, and the existing methods for performance assessment. Then, the methodology section elaborates on the methods. Next, the proposed methods are demonstrated in a case study in Chongqing, China. Finally, a discussion of the findings and critical conclusions are presented.

## 2. Literature Review

### 2.1. The Overall Coordination of Urban Renewal Project on the Area Scale

With the development of urban renewal theory and practice, multi-project planning and management models have gradually attracted academic attention. The importance and significance of the overall coordination of urban renewal projects on the area scale have been well demonstrated. For example, Donaldson and Du Plessis [17] indicated that the benefits of an area-based approach could extend beyond the immediate vicinity and precincts of individual projects and lead to positive spin-offs in a wider range of indirect beneficiaries and communities. Zhu et al. [27] pointed out that the urban recreational business district renewal programs can have a substantial inherent potential to underpin sustainable development and conservation. Liu et al. [15] provided an urban renewal planning and management model to improve land-use efficiency and revitalize the old city via integrated development. It can provide a new view of the balance between resource sustainability, functional adaptability, and cultural continuation during the process of old-city renewal and coordinate the relationship between urban conservation, renewal planning, and industry development. Pérez et al. [14] suggested that the neighborhood scale is the most appropriate for urban renewal planning. At this scale, renewal projects can achieve a larger urban vision and can better adapt to the specificities of the existing buildings, businesses, and inhabitants. In summary, as the frontline for promoting sustainable urban development, the overall coordination of urban renewal projects on the area scale has been increasingly concerning. However, few research studies can provide a clear picture of what urban renewal projects perform well from the area-scale view.

### 2.2. Comprehensive Benefit Evaluation for Urban Renewal Projects

The evaluation of urban renewal sustainability is a complex process [28], with myriad conflicts and trade-offs among various indicators [29]. There is a series of literature addressing the subject of various benefit evaluations for urban renewal projects. The sustainability concept is widely applied in a large number of research studies to conduct benefit evaluation. An indicator-based method, with its sustainability indicators, was conceptualized and developed by utilizing a case study to assess urban renewal sustainability performance [19,21]. Peng et al. [30] integrated a set of sustainability indicators into their developed model to assess the sustainability of urban regeneration. Another indicator-based system, entitled SIPRIUS, was proposed to evaluate sustainability for urban renewal projects, including the project dynamics of regeneration of disused urban areas [31]. Lin et al. [1] adopted a hybrid multiple-attribute decision-making method to evaluate the sustainability of urban renewal projects from the government view. Rooted in the context of Nanjing, China, the benefits of old community renewal were systematically assessed from social, economic, and environmental perspectives [32]. Manupati et al. [33] proposed a comprehensive approach to assess the urban renewal alternative. Korkmaz and Balaban [34] evaluated the sustainability performance of one of the most prominent examples of the squatter regeneration project in Turkey. However, they neglect the fact that in the area scale, different projects can bring added benefits to each other, namely coupling coordination benefits. Even though some studies have mentioned such comprehensive benefits, few can provide a quantitative evaluation [35].

### 2.3. Existing Methods for Project Performance Assessment

It has been recognized that early, continuous, and cautious assessment of project performance is of great importance to improve the quality of project implementation and proposing appropriate strategies [19,20]. Among existing studies, there has been one rich strand of research on the development of the indicator-based evaluation approach. In urban studies, the single indicator-based evaluation approach is the most frequently applied technique in previous research. In particular, fuzzy comprehensive evaluation [36], expert interview [7], case study [37], analytic hierarchy process (AHP) [38], and energy analysis [32] are the most commonly adopted methods. These approaches are mature and easy to conduct. However, these methods are criticized because they cannot accurately accommodate diverse needs for modern decision support [39]. Therefore, some scholars have tried to evaluate the project benefits by combining different methods such as: Monte Carlo simulation and entropy method [30], decision-making trial and evaluation laboratory (DEMATEL) and analytic network process (ANP) [40], DEMATEL-ANP (DANP) and visekriterijumsko kompromisno rangiranje (VIKOR) [1]. Although the integrated assessment model can better deal with problem complexity [39], they all have a common drawback in that the subjective bias cannot be avoided in the data processing.

In summary, existing research studies mainly focus on performance assessment at the single-project level. In addition, the indicators for evaluation commonly include economic, social, and environmental, which refers to the three sustainable dimensions [3]. Few involved coupling coordination benefits among multiple project groups in the area scale. An integrated framework that provides a holistic assessment of area-scale renewal project benefits is missing. Furthermore, the most existent benefit evaluation methods for urban renewal heavily rely on subjective judgment, leading to the deviation of estimation results. To meet the practical demand, the benefit evaluation method for urban renewal should consider overall project coordination on the area scale by introducing a hybrid model to deal with the subjective issue and project complexity.

## 3. Materials and Methods

In this study, a hybrid approach was proposed to quantitatively evaluate the overall benefits of urban renewal projects on the area scale. This research consisted of four main steps. The first step was to build a hierarchical and theory-oriented evaluation indicator system based on the research literature. In the second step, 37 typical area-scale renewal projects in Chongqing were selected, and data were obtained through field survey and expert interviews. Accordingly, the initial weight of each indicator was identified through the entropy weight method. The third step was to develop an artificial neural network based on the back-propagation algorithm to determine the final weight of each indicator. Finally, the comprehensive benefit of the 37 area-scale renewal projects was identified by the linear weighted sum method.

### 3.1. Constructing an Indicator System for Assessing the Comprehensive Benefit

Due to diversification, the process of identifying a series of effective and scientific evaluation indicators is both complex and challenging [5]. The work regarding the combination of sustainable urban renewal and the coupling coordination benefits is quite scarce. However, several studies have provided a good knowledge base from different perspectives, which are summarized in Table 1. Based on the literature review, the benefits of the urban renewal projects on the area scale are identified and categorized into four categories: economic, social and cultural, environmental, and coupling coordination benefits. The details of indicator selection are illustrated as follows:

Whether urban renewal can promote economic sustainability is a vital component in achieving sustainable development. Guaranteeing economic benefits is significant to promote renewal projects continually [7]. Li et al. [32] indicated that the appreciation of fixed assets could be considered as one significant benefit from renewal. The renewal activity can attract more people to rent houses in the community due to the improvement of the living conditions and infrastructure. Meanwhile, some corporations (e.g., housing agencies) can take benefit from the increased rent. Korkmaz and Balaban [34] indicated that urban renewal projects could promote the transformation of the industry and introduce diversified business activities in order to increase regional employment opportunities for local residents and further promote local economic development. Employment can increase the per capita disposable income, which will, in turn, enhance residents’ well-being [34]. Urban renewal faces many cost burdens, including negotiation, relocation, construction, and maintenance costs. Decision-makers will inevitably evaluate the likely return on a given investment [19]. As far as government-led renewal projects are concerned, a limited budget makes it necessary to carefully assess the potential of a project’s resource investment. Therefore, the government-led project is expected to introduce private funds for joint investment, thus reducing the burden of government financial costs. Land redevelopment is a form of resource reuse, and it can optimize land or an individual’s property so that maximum economic benefits are achieved [36]. These indicators are the important driving forces for urban renewal to achieve economic benefits.

It is critical for the social criterion to consider whether a given urban renewal project can promote enhanced quality of life and work in the given area of residence, and especially whether it can benefit vulnerable groups [37]. Providing public infrastructure and public services can improve the quality of services, and thus contributes to promoting the quality of life of different groups in the urban built-up area [3]. Accessibility, the provision of access to various facilities and social services, is a significant form of support for residents [19]. Whether renewal projects can help achieve social sustainability has also risen as a current topic of concern. Zhuang et al. [13] stated that if residents cannot actually benefit from urban renewal and improve social welfare, then the urban renewal will never meet sustainability expectations. Urban renewal can preserve historical structures and characteristics through a design that highlights the region’s uniqueness and protects the heritage and local characteristics [35]. Traditional buildings, as a visually friendly cultural symbol, persuade local residents to pay attention to the uniqueness of their urban environment, which contributes to achieving social sustainability [36]. These indicators are the main driving forces for urban renewal to promote social and cultural benefits.

Given the environmental pressures for urbanization exerts, approaches for achieving environmental sustainability have become a significant strategy for urban renewal projects [1,33]. Environmental benefits are often related to the green space and facilities in urban built-up areas. Greening and open spaces as buffer zones for crowded urban spaces improve residents’ health facilities and their social interactions. Measures such as encouraging the use of green building materials and waste recycling have essential aspects of sustainable development for urban renewal [34]. Design, construction, and maintenance based on environmental sustainability can lead not only to savings with respect to the cost of consuming different renewable and non-renewable materials, but can also help to avoid pollution-related diseases that could cause productivity value loss [29,35]. In the broader context of sustainable development, environmental quality and health are significant attributes of residents’ quality of life [36].

Last but not least, coupling coordination benefits are the significant elements that cannot be neglected for the overall coordination of urban renewal projects on the area scale. Coupling coordination benefits refer to the sharing of manpower, resources, and technology among the renewal projects in the process of multi-project implementation, which generate emerging benefits within the organization, mainly including four aspects: resource utilization, management effects, revenue growth, and target realization [38].

The indicator system reflects the basic elements of the urban renewal performance on the area scale. This considers the rigid need for sustainable urban development; conversely, it also reflects the process of coupling and coordination among the projects under the overall planning of the renewal area.

### 3.2. The Modelling Procedure of the Hybrid Evaluation Model

In order to resolve the existing literature’s shortcomings regarding the comprehensive benefit evaluation, a hybrid assessment model is constructed. The proposed integrated method integrates two different approaches: the entropy weight method and the BP neural network model. First, the entropy weight method is used to identify the initial weight value of each criterion. Then, this initial weight is introduced into the BP neural network to identify the final weight value. The weight value in the hybrid evaluation model indicated the degree of influence of a certain indicator on the comprehensive benefit. According to Lin et al. [49], compared with the traditional single evaluation method, the hybrid approach involves a simple way of formulating an option for an investigation and is more convincing in decision making. The proposed hybrid approach can use each method’s advantages and make up for each other’s deficiencies to reduce the subjective deviation of evaluation results. In general, the modeling procedure includes two main stages as follows:

#### 3.2.1. Entropy Weight Method

The entropy weight method is an objective weighting method that determines the indicator weights based on the information entropy of the indicator. The smaller the information entropy of an element, the greater the degree of the variation. Then, the greater the amount of information provided, the greater the indicator weight. It can capture the implied interactions among indicators and be commonly used to measure value dispersion in decision-making [51]. Where *x* represents the random variable corresponding to the set of all possible outputs, defined as the symbol set, the output of the random variable is expressed as *X*. The *P*(*x*) represents the output probability function, and the greater the uncertainty of the variable, the greater the entropy, and the more information is needed to make it clear.

$$H\left(x\right)=E\left[\log\left(2,\;1/P\left(x_{i}\right)\right)\right]=-\sum P\left(x_{i}\right)\log\left(2,\;P\left(x_{i}\right)\right)\;\left(i=1,2,\cdots ,n\right)\;$$

The weight of each factor can be identified. The steps of identifying the weight by the entropy weight method are summarized as follows [48]:

Step 1: Developing the initial decision matrix as follows:
  $$P=\left[\begin{array}{cccc} p_{11} & p_{12} & \ldots & p_{1n}\\ p_{21} & p_{22} & \ldots & p_{2n}\\ \vdots & \vdots & \vdots & \vdots \\ p_{m1} & p_{m2} & \ldots & p_{mn} \end{array}\right]\;$$
  where *P* is the initial decision matrix, *m* is the number of nodes for benefits evaluation, *n* is the number of elements, *p_ij_* is the evaluation values of each sample parameter, *i* = 0, 1, 2, ⋯, *m*, and *j* = 0, 1, 2, ⋯, *n*.
Step 2: Normalizing the initial decision matrix

It is necessary to normalize the initial decision matrix because the dimension and metric of the data are not uniform. The normalized decision matrix [*Z*] can be expressed as follows:

$$Z=\left[\begin{array}{cccc} z_{11} & z_{12} & \ldots & z_{1n}\\ z_{21} & z_{22} & \ldots & z_{2n}\\ \vdots & \vdots & \vdots & \vdots \\ z_{m1} & z_{m2} & \ldots & z_{mn} \end{array}\right]\;$$

$$\{\begin{array}{l} z_{ij}=\frac{p_{ij}-min\left(p_{ij}\right)}{max\left(P_{ij}\right)-min\left(P_{ij}\right)},\;\;\;\;\left(efficiency\;type\right)\\ z_{ij}=\frac{max\left(p_{ij}\right)-p_{ij}}{max\left(p_{ij}\right)-min\left(p_{ij}\right)},\;\;\;\;\;\;\left(cost\;type\right) \end{array}\;$$

where [*Z*] is the normalized decision matrix.

Step 3: Calculating the entropy value *H_j_* of the *j*-th indicator

The entropy of each element can be calculated as follows:

$$H_{j}=-\frac{\sum_{i=1}^{m}F_{ij}\ln F_{ij}}{\ln m}$$

$$F_{ij}=\frac{z_{ij}}{\sum_{i=1}^{m}z_{ij}}$$

where *H_j_* is the entropy of each element.

Step 4: Calculating the weight *W_j_* of the *j*-th indicator.
  $$W_{j}=\frac{1-H_{j}}{\sum_{j=1}^{n}\left(1-H_{j}\right)}$$
  where *W_j_* is the weight of each element.

The basic properties of entropy weight can be concluded from the above definition. First, if the evaluation value of all evaluation objects is completely the same, the entropy value of the *j*-th indicator is equal to 1, and the entropy weight is equal to 0, which indicates that the indicator does not provide any available information to decision makers and can be considered to remove this indicator, thus the weight of *j*-th is equal to 0. Second, for the *j*-th indicator, if the scale of the object value differs a lot, the smaller the entropy, the larger the entropy weight. This indicates that the *j*-th indicator provides available information to decision makers. In contrast, it also shows that each evaluated object has a large difference and must be focused on.

#### 3.2.2. Back Propagation (BP) Neural Network

The entropy weight method is first used to determine the initial weight of each indicator. Then, this initial weight is introduced into the BP neural network to identify the final weight value. As a type of artificial neural network, BP neural network is a kind of multi-layer feed-forward network based on an error back-propagation algorithm for training, which was proposed by a team of scientists led by Rumelhart and McCelland in 1986. It is widely used to establish an evaluation model and then detect the weights of indicators because of its ability to self-learn, conduct arbitrary function approximation, and seek the optimal solution at high speed [52].

**Figure 1:**
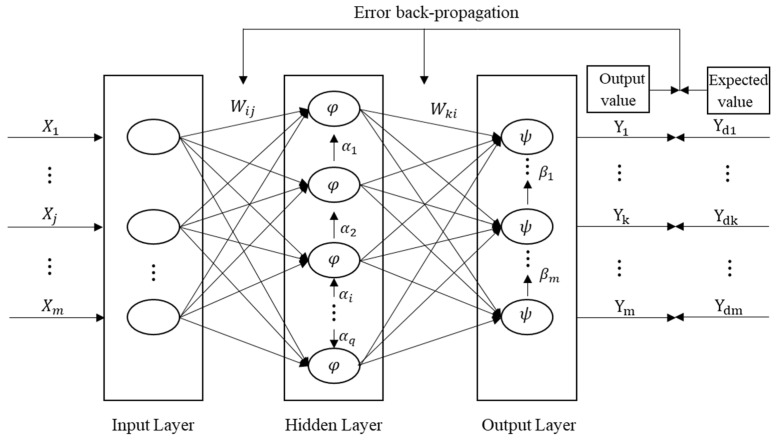
Three-layer BP neural network structure.

As shown in Figure 1, the learning process of the BP neural network includes forward and backward propagation. The signal is transmitted from the input layer to the output layer when the network is in the learning process. However, the gradient is fed back into the network to adjust the weight and bias of each neuron and minimize the error between the output value and expected value if the output results do meet the objective [53].

The objective function of the BP neural network model is expressed as follows [54]:

$$E=\frac{1}{2\times L}\overset{n}{\underset{i=1}{\sum }}\overset{l}{\underset{j=1}{\sum }}\left(y_{ij}-{\hat{y}}_{ij}\right)$$

where *L* is the size of training samples, *l* is the dimension of output variable *y*, and *y_ij_* and ${\hat{y}}_{ij}$ are the output value and expected value, respectively.

The output value of network *y_k_* is defined as follows:

$$y_{k}=f\left(w_{1k}\times o_{1}+\cdots +w_{jk}\times o_{j}+\cdots +w_{nk}\times o_{n}+b_{k}\right)$$

where *f* represents the activation function, *o_j_* is the output value of the *j*-th hidden layer neurons, *w_jk_* is the weights connected with the *j*-th hidden layer neurons and the *k*-th output layer neurons, and *b_k_* is the bias value of the *k*-th output layer neurons.

In order to make the output value close to the expected results, the weights and biases of the network structure need to be updated according to the training error. The updated equation of weights for hidden layers and biases is modified as follows [55]:

$$w_{jk}^{\prime }=w_{jk}-\frac{a}{s}*\overset{s}{\underset{k=1}{\sum }}\Delta k*o_{j}$$

$$b_{j}^{\prime }=b_{j}-\frac{1}{s}*\overset{s}{\underset{k=1}{\sum }}\Delta k$$

where *s* is the neurons number of the output layer, $w_{jk}^{\prime }$ is the updated weights connected with the *j*-th hidden layer neurons and the *k*-th output layer neurons, $b_{j}^{\prime }$ is the updated bias of the *k*-th output layer neurons, and Δ*k* is the training error that us modified as $\Delta k=y_{k}-{\hat{y}}_{k}$.

In the BP neural network developed in this study, the normalized values of 22 evaluation indicators were selected as the input parameters, and the final weights of the 22 indicators were applied as the output parameters. Meanwhile, the activation function of the hidden layer adopted the Tansig function, as shown in the following equation:

$$f\left(x\right)=\frac{2}{1+exp\left(-2x\right)}-1$$

A linear activation function, namely the Purelin function, was used as the activation function of the output layer for transferring from the hidden layer to the output layer:

f(x)=x

In this study, 22 samples of initial weights generated in the entropy weight method were randomly divided into three groups for training, validation, and testing of the BP neural network. After that, the most important step is to identify the number of neurons in the hidden layer, which can be calculated by using the following equation:

$$N=\sqrt{m+l}+a$$

Where *N* is the number of neurons in the hidden layer, *m* is the number of neurons in the input layer, *l* is the number of neurons in the output layer, and *a* represents the constant between 1 and 10.

Finally, mean square error (MSE), mean absolute error (MAE), mean absolute percentage error (MAPE), and determination coefficient (*R*^2^) are adopted to evaluate the model accuracy [56]. By adjusting the number of neuron nodes and multiple training, the result with smaller error indicators and better *R*^2^ was chosen.

$$MSE=\frac{\sum_{i=1}^{n}{\left(x_{output,i.j}-x_{target,i.j}\right)}^{2}}{n}$$

$$MAE=\frac{1}{n}\overset{n}{\underset{i=1}{\sum }}|\frac{{\hat{y}}_{i}-y_{i}}{y_{i}}|$$

$$MAPE=\frac{100\%}{n}\overset{n}{\underset{i=1}{\sum }}\frac{{\hat{y}}_{i}-y_{i}}{y_{i}}\;R^{2}=1-\frac{\sum_{i=1}^{n}{\left(x_{output,i.j}-x_{target,i.j}\right)}^{2}}{\sum_{i=1}^{n}{\left(x_{target,i.j}-{\bar{x}}_{target,i.j}\right)}^{2}}$$

where *n* is the number of the testing group, *x_output,i.j_* is the estimated value, *x_target,i.j_* is the actual value, ${\bar{x}}_{target,i.j}$ is the average value of *x_target,i.j_*, and *k* is the number of model parameters.

#### 3.2.3. Linear Weighted Sum Method

One of the main ways of weighting methods is the linear weighted sum method. It is an efficient approach for assessing comprehensive benefits by quantifying the benefit as a score. This implies that an area-scale renewal project with a higher score could have a better comprehensive benefit. The purpose of the linear weighted sum method is to quantify the benefit of different area-scale renewal projects and to distinguish the levels of comprehensive benefits. In the model of this method, sum weighting coefficient *w_i_* and multiply each evaluation value of each indicator *x_ij_* to make the structure of the formula as follows:

$$g_{j}=\overset{n}{\underset{i=1}{\sum }}w_{i}*x_{ij}$$

where $w_{i}\geq 0;\;\overset{n}{\underset{i=1}{\sum }}w_{i}=1$; *x_ij_* is the standardized value of the *i*-th indicator in the *j*-th area-based renewal initiative; *w_i_* is the coefficient of *i*-th indicator.

## 4. Case Study and Results

### 4.1. Case Background

Located in Southwest China, Chongqing is a typical high-density city with a territory of approximately 82,400,000,000 m^2^, containing nearly 32,050,000 residents [57]. Its urban development has faced physical, environmental, and social problems such as building dilapidation, environmental deterioration, and traffic congestion. As these challenges in Chongqing become increasingly prominent, undertaking urban renewal projects to improve living conditions and the urban environment is imminently needed [29]. Therefore, plenty of urban renewal projects have been implemented in recent years. From 2018 to 2022, Chongqing continued to promote 112 demonstration projects for urban renewal, with a total investment of approximately 167.5 billion yuan. Therefore, Chongqing could provide abundant cases and resources for urban renewal studies [13]. In this study, 37 urban renewal projects on the area scale were selected as the typical case of comprehensive benefit evaluations.

### 4.2. Data Collection

In order to determine the influence degree of the 22 indicators that have been identified above, a seminar was organized at Chongqing university. Because the hybrid approach proposed in this study belongs to the decision-making method for experts, a sizable sample is not required [58]. The group was composed of five professional experts in the field of urban renewal and three project managers operating multiple renewal projects. An exclusive expert decision-making questionnaire designed in Chinese was first developed for data collection. The interviewed experts mainly determined the case’s potential for comprehensive benefits for 22 indicators that were proposed through an equidistant 5-point evaluation scale (1 = little impact to 5 = high impact).

### 4.3. Results with Entropy Weight Method

In the evaluation of comprehensive benefits for area-scale renewal projects, the relative importance of each indicator is different, so the weight distribution of each indicator should be identified first to conduct the BP neural network of indicators. The original data obtained by the questionnaire were sorted out, and the weighting relationships of indicators were identified by the entropy weight method. It should be noted that the indicator system constructed in this study is hierarchical. When determining the initial weights, it is necessary to calculate the weights of each indicator first and then obtain the four criteria by utilizing the weighted arithmetic square method combined with the coefficient of the four criteria, and then identify the weight of each criterion by using the entropy weight method. The scoring results of eight experts on the benefit evaluation of 37 area-scale renewal projects are summarized and substituted into the above-mentioned formulae, and the weights of each indicator are calculated, as shown in Table 2.

In summary, the initial weight values of the entropy method of each indicator under the four dimensions of economic benefit, social and cultural benefit, environmental benefit, and coupling coordination benefit have been calculated. Then, the initial evaluation value of the comprehensive benefits of 37 area-scale renewal projects is determined by the linear weighted sum method, as summarized in Table 3.

### 4.4. Results of Final Comprehensive Benefits

Four BP neural network models are constructed by MATLAB 2021 software, and the evaluation objects are economic benefits, social and cultural benefits, environmental benefits, and coupling coordination benefits, respectively. The corresponding network topology results are summarized as follows: 8 × 10 × 1, 6 × 10 × 1, 4 × 10 × 1, 4 × 10 × 1. The 34 groups of data were used as training samples, and the 3 groups of data were randomly selected as test samples from Table 3. The training times were set as 1000, the target error was set as 0.05, the learning rate was set as 0.1, and then the network was trained to converge to complete the BP neural network construction process. The previous study indicated that repeated trials and experiments and controlling the error could improve the output [52,57,59]. Meanwhile, in general, after undergoing an increasing number of training stages, the error is reduced. In this study, the accuracy of the BP neural network was improved by increasing the number of training times and controlling the error rate.

**Figure 2:**
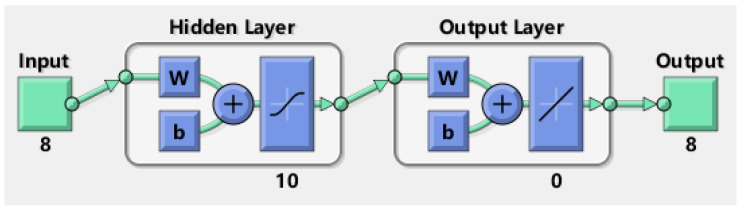
The BP neural network model of economic benefit dimension.

The standardized evaluation value and the initial indicator weight value are taken as the input of the BP neural network, and the final indicator weight value is used as the output. Taking the economic benefit dimension as an example, run the MATLAB 2021 software to obtain the BP neural network model. The BP neural network model under the dimension of economic benefit is elaborated as shown in Figure 2.

**Figure 3:**
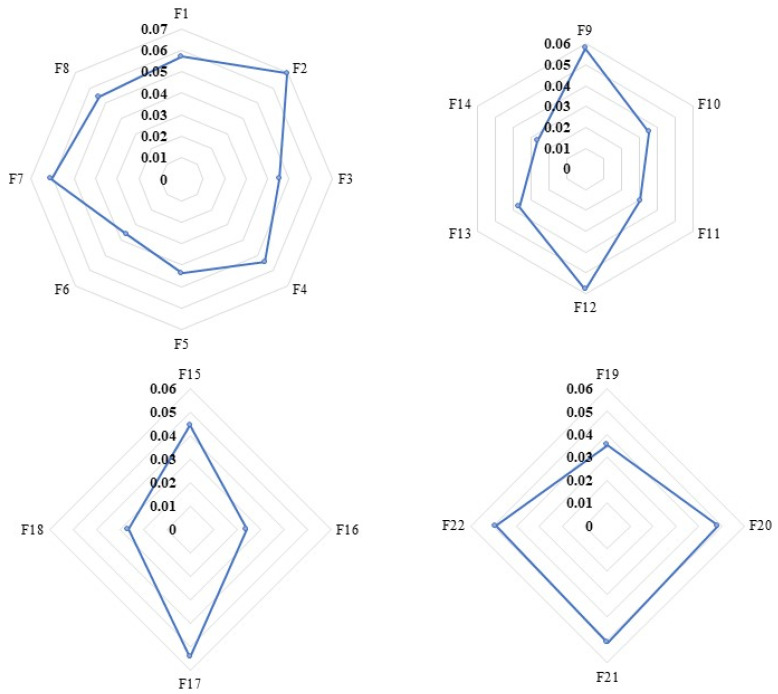
The final weights of each indicator.

Through MATLAB 2021 software simulation, BP neural network can achieve better accuracy after training, input three groups of test samples to update relevant data, ensure that the error is within the set range, and meet the requirements of inspection and evaluation so that the results are more objective and accurate. In particular, the coefficient value of the error indicators MSE, MAE, and MAPE are 0.026097, 0.086272, and 2.939%, respectively. In addition, the indicator training results indicated that the BP neural network had a better linear regression, with a determination coefficient *R*^2^ of 0.91088. The results showed that the errors are within the control range, and the experimental results are satisfactory. Therefore, this BP neural network has strong applicability in determining the final weight value. The final weight value of the BP neural network, the initial weight value of the entropy method, and the error of each indicator under the four dimensions of economic benefit, social and cultural benefit, environmental benefit, and coupling coordination benefit were obtained, which are summarized respectively in Table 4 and are elaborated in Figure 3.

In summary, through the BP neural network model, the final weight value of the evaluation indicator under each dimension is obtained, and the error is within the control range, indicating that the operation effect of the model is satisfactory. Thus, the BP neural network can optimize the results of the entropy weight method to make its indicator weight results more accurate. The final weight value of the indicator obtained by the BP neural network is used to calculate the final comprehensive benefit values of the renewal initiatives on the area scale, which are summarized in Table 5.

**Figure 4:**
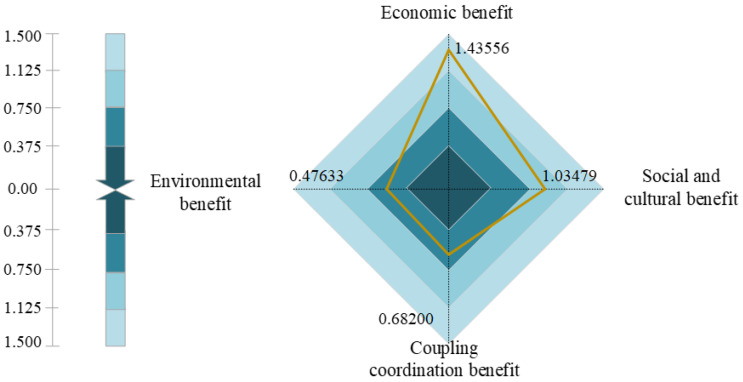
The comprehensive benefits of SPBSB area-scale renewal projects.

In order to present each component of comprehensive benefits for the coordination of urban renewal projects in the area scale more clearly, this study takes N20 as a typical example and plots the benefit value of each dimension in the front of the radar chart, as shown in Figure 4. It can be found that the order of benefit value from high to low is an economic benefit, social and cultural benefit, coupling coordination benefit, and environmental benefit. In addition, the coupling coordination benefit accounts for approximately 19% of the total benefit and is an important portion of the comprehensive benefit.

## 5. Discussion

### 5.1. Benefit Evaluation of Urban Renewal Projects on the Area Scale Based on the Entropy Weight and BP Neural Network Method

This research indicated that the entropy weight method and BP neural network method have obvious complementarity. The combination of these two methods provides insight into the benefit evaluation of urban renewal projects on the area scale. The entropy weight method is an effective tool in indicator weight determination. The initial weights of each indicator were identified based on the entropy weight method. However, due to the lack of comparison between indicators, the entropy weight method has its drawbacks in data analysis and processing in practical applications. In addition, since the weight value of each indicator depends on the selected sample, the results may include deviations at a certain level. Therefore, the initial weight was processed further through the learning mechanism of the BP neural network model. It can reduce the error caused by the entropy weight method and make the result more scientific and objective.

In addition, compared to the existing studies, the set of indicators selected in this research can be appropriately used for performance assessment of urban renewal projects on the area scale. Evaluating the urban renewal project performance considering the overall coordination on the area scale is necessary and significant for the decision support in modern sustainable urban development [5]. Many research studies have evaluated urban renewal projects from economic, social, and environmental aspects to meet the requirement of sustainability. On this basis, this study further takes into account the coupling coordination benefits among the projects from the perspective of overall coordination in project management. The indicator of coupling coordination benefits can reflect the sharing of manpower, resources, and technology among several projects in the process of multi-project implementation.

The integrated use of the entropy weight and BP neural network method has brought about added benefits, in particular by providing a suitable evaluation tool for an indicator-based approach. The results give not only a holistic picture of the comprehensive benefits of urban renewal projects on the area scale but also the direction for future improvement. In addition, the hybrid approach can provide insights for performance evaluation in other urban development issues.

### 5.2. Differences in Area-Scale Renewal Project Performances

This hybrid approach quantified the comprehensive benefits of urban renewal on the area scale. The results showed that the overall comprehensive project performances in the case area were at a relatively moderate level. The maximum total performance score of the sampling initiative was 3.98939, while the lowest was 2.69287. The maximum score was over 1.5 times higher than the minimum one, which indicated a significant difference in performance between these urban renewal initiatives. In detail, N7 performed best in the study area. It ranked well in economic, social and cultural, and coupling coordination dimensions but produced a lower score in environmental aspects. This was mainly due to the increasing rental incomes (*F*_2_), higher providing jobs for residents (*F*_12_), and coupled synergies in management effects (*F*_21_). Although the comprehensive benefit value of N7 ranks first, its increasing greening and open space (*F*_16_) was not ideal. N10 presented the poorest comprehensive benefit. The result shows its drawbacks from all dimensions. This may be due to its complicated project situation, including development orientation, management mode, and external environment [5]. The overall results indicated that many projects fail to meet the requirement of sustainability and overall coordination in urban renewal initiatives.

The results can also provide references for policy-makers for specific overall coordination of urban renewal projects in the area scale through quantified measurement. It depicts a clear picture of the project performance in comprehensive dimensions. For instance, it clearly shows that N20 performed well in the economic aspect, which means it can contribute greatly to regional economic growth. However, its low environmental benefit clearly reveals the ignorance of this aspect during its planning and implementation process. Specifically, both the improvement of environmental quality and greening and open space need to receive greater attention.

### 5.3. Improvement Strategies/Suggestions for Future Area-Scale Renewal Projects

This research shed light on proposing improvement strategies for urban renewal on the area-scale scale. These strategies lie in two critical dimensions, which are sustainability and the coupling coordination of projects.

Urban planners and policymakers should first pay attention to the elements/indicators that own relatively small weights as well as those with poor overall project performance. Especially, reducing the financial cost burden (*F*_6_), inheriting historical and cultural values and city style (*F*_14_), and increasing greening and open space (*F*_16_) can be regarded as comprising the critical improvement core in the sustainable dimensions. Regarding improving land-use efficiency, the mixed-use of urban land should be strengthened, and the process should involve rationalizing the functional allocation and carrying out elastic control. In addition, due to the cultural dimension’s poor performance, urban planners and policymakers need to consider how to integrate the renewal projects with more local culture, such as integrating building heritage conservation and tourism area construction. The introduction of artistic and cultural activities incorporating heritage routes will also promote interaction between the community and visitors [15,60]. Improving environmental quality through green and open space and coordinated urban design can connect the existing open space system in keeping with the planning concept, construct the regional image, and improve the quality of the environment and landscape [1,61].

In contrast, to better promote urban renewal projects, it is necessary to break through the management boundary and combine the multiple projects appropriately. Due to the spatial fragmentation of the current situation, urban renewal projects are often trapped in an isolated planning and management mode, posing financial barriers for low-profit or unprofitable projects and causing ratcheting tensions in human–land relationships. Therefore, shifting the traditional idea to the overall coordination in the area scale is of great significance in addressing the issue of urban renewal projects. Moreover, compared with single project implementation, it is argued that the advantages of the overall coordination of urban renewal projects in the area scale can be well reflected in the aspects of cost-saving, profit increase, and efficiency improvement [15]. In recent years, project portfolio management theory has attracted increasing attention in the field of project management. It strongly matches the characteristics of urban renewal, and can be used to plan and manage renewal projects in an area scope appropriately.

## 6. Conclusions

In order to achieve sustainable urban development, the overall coordination of urban renewal projects on the area scale has been advocated globally. Planning and initiating urban renewal from a broader area perspective can better promote the projects and bring about comprehensive benefits to urban built-up areas [62,63]. However, it still lacks a systematic evaluation framework to obtain a clear picture of such benefits. To meet such practical demand, this study proposed an integrated framework for evaluating the comprehensive benefits of area-scale renewal projects, covers four criteria and 22 indicators, provides a broader understanding with respect to evaluating benefits more objectively based on entropy weight and BP neural network, and eliminates the shortcoming of the traditional assessment method. This study takes 37 area-scale renewal projects in Chongqing as a study area. First, it constructs an integrated indicator-based framework that includes economic, social and cultural, environmental, and coupling coordination criteria. Second, the entropy weight method was used to identify the initial weights of each indicator. Finally, the BP neural network was applied to determine the final weights, and then the comprehensive benefits of 37 area-scale renewal projects were calculated. This study also revealed the necessity of adopting the overall coordination mode of urban renewal and helping urban planners and policymakers to understand the importance of sustainable development. Moreover, this research facilitates the development of knowledge in the discipline of traditional project management theory, which can also be used to guide benefit evaluation in practice.

Although this research has both critical, practical, and academic significance, there are also some limitations. Chongqing is a typical city in China and can represent a large part of urban development characteristics. However, one city cannot represent every case. There are still some cities with specific urban development types and periods. These cities may require different urban renewal performances, which is worth studying. Future studies can use more cases by considering the characteristics of different cities and periods of urban development to reveal the full picture of global urban renewal practices.

## Figures and Tables

**Table 1:** Indicators for comprehensive benefit evaluation of urban renewal projects on the area scale.

Criteria	Indicator	Reference
Economic benefits	Appreciation of the fixed assets (*F*_1_)	[23,32,36,39]
	Increasing the rental income (*F*_2_)	[14,23,33,36]
	Improving the corporate reputation and revenue (*F*_3_)	[32,40]
	Increasing the fiscal revenue from land (*F*_4_)	[39,40,41]
	Promoting the local economic development (*F*_5_)	[1,23,33,36,40,42,43,44]
	Reducing the financial cost burden (*F*_6_)	[1,14,36]
	Increasing the per capita disposable income (*F*_7_)	[40,45]
	Improving land-use efficiency (*F*_8_)	[1,14,39,40,43]
Social and cultural benefits	Improving the public infrastructure (*F*_9_)	[14,23,33,39,40,41,43,44,45,46]
	Strengthening the accessibility of public facilities (*F*_10_)	[1,14,23,33,34,39,41,44,45,46]
	Enhancing cultural and educational attainment (*F*_11_)	[14,33,36,41,46]
	Providing jobs for residents (*F*_12_)	[14,23,33,34,36,39,41,43,44,45,46,47]
	Promoting the social welfare (*F*_13_)	[1,33,35,45,48,49]
	Inheriting historical and cultural values and city style (*F*_14_)	[1,14,36,40,42,43,44,45,46]
Environmental benefits	Improving environmental quality (*F*_15_)	[1,3,20,29,41,43,44,45,48,49,50]
	Increasing greening and open space (*F*_16_)	[1,14,32,33,34,39,41,43,44,45,46]
	Using green building materials (*F*_17_)	[14,34,41,43,44,45]
	Reducing building energy consumption levels (*F*_18_)	[1,16,32,34,36,41,43,44,45,46,47]
Coupling coordination benefits	Coupled synergies in revenue growth (*F*_19_)	[25,50]
	Coupled synergies in resource utilization (*F*_20_)	[25,26,50]
	Coupled synergies in management effects (*F*_21_)	[14,25,45,50]
	Coupled synergies in target realization (*F*_22_)	[14,25,26,45,46,50]

**Table 2:** The initial weights of each indicator.

Criterion	Indicator	Weight
Economic benefits	Appreciation of the fixed assets (*F*_1_)	0.03463
	Increasing the rental income (*F*_2_)	0.08091
	Improving the corporate reputation and revenue (*F*_3_)	0.09549
	Increasing the fiscal revenue from land (*F*_4_)	0.09568
	Promoting the local economic development (*F*_5_)	0.05203
	Reducing the financial cost burden (*F*_6_)	0.01894
	Increasing the per capita disposable income (*F*_7_)	0.05975
	Improving land-use efficiency (*F*_8_)	0.01889
Social and cultural benefits	Improving the public infrastructure (*F*_9_)	0.07851
	Strengthening the accessibility of public facilities (*F*_10_)	0.01700
	Enhancing cultural and educational attainment (*F*_11_)	0.04447
	Providing jobs for residents (*F*_12_)	0.02304
	Promoting the social welfare (*F*_13_)	0.02382
	Inheriting historical and cultural values and city style (*F*_14_)	0.01396
Environmental benefits	Improving environmental quality (*F*_15_)	0.01889
	Increasing greening and open space (*F*_16_)	0.01883
	Using green building materials (*F*_17_)	0.11832
	Reducing building energy consumption levels (*F*_18_)	0.10010
Coupling coordination benefits	Coupled synergies in revenue growth (*F*_19_)	0.01937
	Coupled synergies in resource utilization (*F*_20_)	0.03356
	Coupled synergies in management effects (*F*_21_)	0.02304
	Coupled synergies in target realization (*F*_22_)	0.01076

**Table 3:** The initial evaluation value of comprehensive benefit for 37 area-scale renewal projects.

No	Area-Scale Renewal Projects	Comprehensive Benefits
N1	YPJ1862 area-scale urban renewal projects	2.55842
N2	CQPX area-scale urban renewal projects	3.10497
N3	CQK area-scale urban renewal projects	2.82352
N4	JZS area-scale urban renewal projects	2.44840
N5	LMH area-scale urban renewal projects	3.10080
N6	SBT area-scale urban renewal initiative	3.20423
N7	ELEC area-scale urban renewal projects	3.63145
N8	CYJ area-scale urban renewal projects	3.50702
N9	JLJ area-scale urban renewal projects	3.04263
N10	XJL 24th area-scale urban renewal initiative	2.34403
N11	CGC area-scale urban renewal projects	2.60960
N12	JLP area-scale urban renewal projects	3.94915
N13	JBZJ-TGC area-scale urban renewal projects	2.98495
N14	DYM area-scale urban renewal projects	3.82974
N15	SYL area-scale urban renewal initiative	2.72866
N16	SZJ-WGT area-scale urban renewal projects	3.28858
N17	MZC area-scale urban renewal projects	3.16128
N18	GJTWJ area-scale urban renewal projects	2.75477
N19	LWX area-scale organic renewal projects	3.38503
N20	SPBSB area-scale urban renewal projects	3.22482
N21	CQYK area-scale urban renewal projects	2.79377
N22	XJW area-scale urban renewal projects	2.70096
N23	DPZJ area-scale urban renewal projects	3.45692
N24	MJB area-scale urban renewal initiative	3.71184
N25	FTG area-scale urban renewal projects	3.40208
N26	CYB area-scale urban renewal projects	3.35363
N27	RMC area-scale urban renewal projects	3.31712
N28	RHJD area-scale urban renewal initiative	3.31968
N29	HQB area-scale urban renewal projects	3.38989
N30	JTJ area-scale urban renewal projects	3.53199
N31	JYW area-scale urban renewal projects	2.92998
N32	LZB area-scale urban renewal projects	3.65634
N33	GHYXC area-scale urban renewal initiative	3.46880
N34	JXC area-scale urban renewal projects	3.23006
N35	LJM area-scale urban renewal projects	3.36210
N36	GYQ area-scale urban renewal initiative	3.52166
N37	YSG area-scale urban renewal projects	3.66296

**Table 4:** The final weights of each indicator.

Criterion	Indicator	Initial Weight	Final Weight	Error
Economic benefits	Appreciation of the fixed assets (*F*_1_)	0.03463	0.05698	0.00434
	Increasing the rental income (*F*_2_)	0.08091	0.06959	
	Improving the corporate reputation and revenue (*F*_3_)	0.09549	0.04543	
	Increasing the fiscal revenue from land (*F*_4_)	0.09568	0.05466	
	Promoting the local economic development (*F*_5_)	0.05203	0.04384	
	Reducing the financial cost burden (*F*_6_)	0.01894	0.03621	
	Increasing the per capita disposable income (*F*_7_)	0.05975	0.06079	
	Improving land-use efficiency (*F*_8_)	0.01889	0.05412	
Social and cultural benefits	Improving the public infrastructure (*F*_9_)	0.07851	0.05783	0.00734
	Strengthening the accessibility of public facilities (*F*_10_)	0.01700	0.03535	
	Enhancing cultural and educational attainment (*F*_11_)	0.04447	0.03060	
	Providing jobs for residents (*F*_12_)	0.02304	0.05793	
	Promoting the social welfare (*F*_13_)	0.02382	0.03666	
	Inheriting historical and cultural values and city style (*F*_14_)	0.01396	0.02646	
Environmental benefits	Improving environmental quality (*F*_15_)	0.01889	0.04432	0.02665
	Increasing greening and open space (*F*_16_)	0.01883	0.02404	
	Using green building materials (*F*_17_)	0.11832	0.05449	
	Reducing building energy consumption levels (*F*_18_)	0.10010	0.02668	
Coupling coordination benefits	Coupled synergies in revenue growth (*F*_19_)	0.01937	0.03532	0.02432
	Coupled synergies in resource utilization (*F*_20_)	0.03356	0.04841	
	Coupled synergies in management effects (*F*_21_)	0.02304	0.05128	
	Coupled synergies in target realization (*F*_22_)	0.01076	0.04901	

**Table 5:** The final evaluation value of comprehensive benefit.

No	Area-Scale Renewal Projects	Comprehensive Benefits
N1	YPJ1862 area-scale urban renewal projects	3.01862
N2	CQPX area-scale urban renewal projects	3.50265
N3	CQK area-scale urban renewal projects	3.33118
N4	JZS area-scale urban renewal projects	2.98858
N5	LMH area-scale urban renewal projects	3.26097
N6	SBT area-scale urban renewal initiative	3.55709
N7	ELEC area-scale urban renewal projects	3.98939
N8	CYJ area-scale urban renewal projects	3.71691
N9	JLJ area-scale urban renewal projects	3.43270
N10	XJL 24th area-scale urban renewal initiative	2.69287
N11	CGC area-scale urban renewal projects	2.85585
N12	JLP area-scale urban renewal projects	3.59514
N13	JBZJ-TGC area-scale urban renewal projects	3.27931
N14	DYM area-scale urban renewal projects	3.83761
N15	SYL area-scale urban renewal initiative	3.11313
N16	SZJ-WGT area-scale urban renewal projects	3.74100
N17	MZC area-scale urban renewal projects	3.53106
N18	GJTWJ area-scale urban renewal projects	2.82915
N19	LWX area-scale organic renewal projects	3.38690
N20	SPBSB area-scale urban renewal projects	3.34398
N21	CQYK area-scale urban renewal projects	2.93988
N22	XJW area-scale urban renewal projects	2.84704
N23	DPZJ area-scale urban renewal projects	3.26779
N24	MJB area-scale urban renewal initiative	3.62822
N25	FTG area-scale urban renewal projects	3.57984
N26	CYB area-scale urban renewal projects	3.18215
N27	RMC area-scale urban renewal projects	3.17874
N28	RHJD area-scale urban renewal initiative	3.63834
N29	HQB area-scale urban renewal projects	3.45906
N30	JTJ area-scale urban renewal projects	3.52328
N31	JYW area-scale urban renewal projects	3.20014
N32	LZB area-scale urban renewal projects	3.61487
N33	GHYXC area-scale urban renewal initiative	3.57732
N34	JXC area-scale urban renewal projects	3.44440
N35	LJM area-scale urban renewal projects	3.37760
N36	GYQ area-scale urban renewal initiative	3.61989
N37	YSG area-scale urban renewal projects	3.87100

## Data Availability

All data collected during the study are available in the submitted article and the detailed data values are available from the corresponding author by request.
